# Understanding the Impact of Communicating Uncertainty About COVID-19 in the News: Randomized Between-Subjects Factorial Experiment

**DOI:** 10.2196/51910

**Published:** 2024-05-14

**Authors:** Rui Zhao, Xuerong Lu, Jiayi Yang, Biao Li

**Affiliations:** 1 School of Journalism and Communication Minzu University of China Beijing China; 2 School of Communication Oregon State University Corvallis, OR United States; 3 School of Chinese Language and Literature University of International Business and Economics Beijing China; 4 School of Journalism Renmin University of China Beijing China

**Keywords:** information uncertainty, health communication, uncertainty management, COVID-19, public health perception, health information

## Abstract

**Background:**

Whether and how the uncertainty about a public health crisis should be communicated to the general public have been important and yet unanswered questions arising over the past few years. As the most threatening contemporary public health crisis, the COVID-19 pandemic has renewed interest in these unresolved issues by both academic scholars and public health practitioners.

**Objective:**

The aim of this study was to investigate the impact of communicating uncertainty about COVID-19–related threats and solutions on individuals’ risk perceptions and misinformation vulnerability, as well as the sequential impact of these effects on health information processing and preventative behavioral intentions.

**Methods:**

A 2×2 (threat uncertainty [presence vs absence]×solution uncertainty [presence vs absence]) full-fractional between-subjects online experiment was conducted with 371 Chinese adults. Focusing on the discussion of whether the asymptomatic cases detected during the COVID-19 pandemic would further lead to an uncontrolled pandemic, news articles were manipulated in terms of whether the infectiousness of asymptomatic cases and the means to control the transmission are presented in terms of their certainty or uncertainty. Participants were randomly assigned to one of the four experimental conditions, being instructed to read one news article. After reading the news article assigned, participants were asked to respond to a series of questions to assess their cognitive and behavioral responses.

**Results:**

Individuals were more susceptible to believing false COVID-19–related information when a certain threat and uncertain solution were framed in the news article. Moreover, individuals’ perceptions of crisis severity increased when exposed to news information containing uncertain solutions. Both misinformation vulnerability and perceived severity were positively associated with information processing. Information seeking was positively associated with protective behavioral intention, whereas information avoidance was negatively associated with protective behavioral intention.

**Conclusions:**

Our findings imply that uncertainty, depending on its aspect, can be effectively communicated to the public during an emerging public health crisis. These results have theoretical and practical implications for health communicators and journalists. Given its limited influence on individuals’ cognitive and behavioral responses, uncertainty related to a health threat should be disseminated to meet the public’s expectation of information transparency. However, caution is advised when communicating uncertainty related to potential solutions, as this factor exhibited a mixed impact on individual responses during a crisis.

## Introduction

### Background

The question of how to communicate uncertainty to the general public has been raked up during the COVID-19 pandemic, which is considered to be the most threatening public health crisis that emerged over the past 10 years, characterized by a high level of uncertainty. Since its outbreak, news coverage of COVID-19 has largely been emphasizing the “unknowns” about the source, infectivity, treatment, prevention, and control measures of the virus [1]. However, whether (or not) uncertainties should be communicated to the general public remains a controversial issue, given the general low tolerance of the public for uncertainty along with a high expectation for information transparency. On the one hand, uncertainty is an undesirable experience in which people fear losing control of their lives, leading to negative consequences [2,3]. On the other hand, uncertainty may also have positive effects, as some scholars suggest that when uncertainty is perceived, people tend to actively seek for information to ease this feeling, and in this process can gain more information and a deeper understanding of the event [4]. Thus, it is important to understand whether the uncertainty presented in news articles influences individuals’ cognitive and behavioral intentions during public health emergencies.

In actual news framing, uncertainty does not appear as a monolithic entity, and each new challenge presented by COVID-19 involves different aspects (eg, threats and solutions) with varying degrees of uncertainty [5]. According to the Centers for Disease Control and Prevention, the uncertainties related to threats and solutions are the two greatest concerns among the public during public health emergencies [6]. Therefore, this study focused on the uncertainties related to threats and solutions associated with COVID-19 that the news media might (or might not) communicate to the public, with the goal of exploring how this communication of uncertainty might influence individuals’ health behaviors.

### Impacts of Uncertainty on Risk Perception and Misinformation Vulnerability

Risk perception is always associated with uncertainty in the public health context. It is assumed that individuals will only begin to manage uncertainty through information processing or preventive behaviors when they perceive a given issue to be associated with a certain level of risk [7]. The perceived risk by individuals includes the severity and susceptibility of a public health crisis. Severity refers to the magnitude of harm caused by the threat, whereas susceptibility refers to the probability of occurrence of a threat to a specific subject [8]. Empirically, Lalot et al [9] found that individuals’ uncertainty about how the novel coronavirus would affect people significantly increased their perceived threat of the pandemic. Pine et al [10] found that the partial and changing information would cultivate the uncertainty surrounding COVID-19, which would further influence individuals’ risk perception of the pandemic.

Moreover, the potential impact of uncertainty on exacerbating the misinformation effect has been raised as a concern in recent years. Lu et al [11] found that communicating uncertainty about preliminary evidence related to COVID-19 was positively associated with the number of likes and retweets of related misinformation on social media. Consequently, the communication of uncertainty during a pandemic might unexpectedly facilitate engagement with misinformation.

To better understand the impact of threat uncertainty and solution uncertainty that are communicated to the general public during a public health crisis, we established the following research question: How, if at all, does the uncertainty frame of a threat and solution exert main and interaction effects on individuals’ risk perceptions and misinformation vulnerability?

### Impacts of Uncertainty on Information Processing

Information seeking is regarded as a key communication outcome during a public health crisis, guiding individuals to understand public health issues and consequently adopt appropriate health behaviors (eg, [7,12]). Information seeking refers to individuals’ active information-searching activity through human interaction [13], online inquiry [14], and passive observation [15]. Theoretical and empirical evidence suggests that individuals tend to engage in information-seeking behaviors when experiencing psychological discomfort such as confusion and anxiety resulting from exposure to uncertainty (eg, [16,17]). Although the motivations for information-seeking behaviors according to various demographic characteristics such as age, gender, and health status have been extensively examined in the public health context (eg, [12,18]), little is known about how uncertainty communicated by the media and experts influences individuals’ information-seeking behavior.

Case et al [19] pointed out that individuals might also engage in information avoidance to reduce feelings of uncertainty. They found that the mental discomfort that arises due to uncertainty, especially in a health context, could facilitate information-avoidance behaviors. Information avoidance refers to an individual’s absence from or ignorance of information and its source [4].

Thus, we further aimed to understand whether (or not) and how the uncertainty framed in the news would motivate individuals’ information-seeking and information-avoiding behavior differently.

Additionally, the relationship between risk perception and information processing has been documented in the health risk literature. Goodall and Reed [20] suggested that individuals would seek more information when the perceived threat is high. Conversely, Jiang et al [21] found that a higher risk perception of the COVID-19 pandemic would reduce individuals’ information-seeking behavior. They also found that individuals’ propensity to believe COVID-19 misinformation would also influence their additional information-seeking behavior.

Therefore, we sought to examine how, if at all, uncertainty framed in severity and susceptibility influences information processing (ie, information seeking and information avoidance) through risk perceptions and misinformation vulnerability.

### Information Seeking and Avoidance Influence Preventive Behaviors

In the context of COVID-19, studies on how organizations and individuals perceive risks during crises have centered on the changes in preventive behaviors during infectious disease outbreaks and how these behavioral shifts can be facilitated by engagement in informational behaviors.

Previous studies suggest that information seeking through different channels and sources is positively associated with preventive behaviors during crises (eg, [22,23]). Individuals who engage in more effortful information seeking and processing are more likely to develop risk-related cognitions, attitudes, and behaviors [24]. However, the health information environment in a pandemic is often filled with uncertain information, false claims, or even conspiracy theories [25], which can bias people’s pandemic-related beliefs and impede their adoption of effective actions [26].

Nevertheless, this situation does not imply that refraining from active information seeking is a wise choice. By contrast, the impact of information avoidance on preventive behaviors is not less significant than that of information seeking [27]. While information avoidance minimizes the chances of interaction with unnecessary information, it simultaneously diminishes the opportunities to receive relevant information. From a cognitive perspective, individuals have limited capacity to process information, and if not adequately addressed, the outcome can be information overload [28]. Avoiding information acquisition may lead individuals to make preventive decisions based on limited information [29]. Particularly when faced with uncertain information, information avoidance may lead to incorrect preventive behaviors. Therefore, we sought to determine how, if at all, preventative behavioral intentions might be associated with information seeking (1) and information avoidance (2) separately.

## Methods

### Research Design

This study adopted a controlled experiment approach. Based on the question “*Will asymptomatic cases lead to an uncontrolled epidemic?*” a 2×2 (threat certainty vs uncertainty×solution certainty vs uncertainty) online experiment was designed using asymptomatic cases, an emerging challenge in the COVID-19 epidemic, as a risk scenario. Data were collected in May 2020 through this anonymous online experiment. Participants were recruited from Sojump, which is the largest online survey platform in China. As the context of the experiment, at this time, China was gradually implementing measures to prevent and control the COVID-19 epidemic. However, at the same time, the detection of an increasing number of asymptomatic cases was raising concern. On March 31, 2020, China’s National Health Commission announced that as of the following day (April 1), it would disclose the detection, transition, and management of asymptomatic cases in its daily briefings on the epidemic to respond to these societal concerns in a timely manner [30]. The risk threat posed by the emerging challenge and COVID-19 prevention measures were still being explored and discussed at that time and were fraught with uncertainties. Based on this context, we developed 4 simulated online news reports based on real news coverage and expert interview data; the details of the simulations are provided in Multimedia Appendix 1 with a brief summary in Table 1.

Although the content orientation of the simulated news coverage differed across the 4 scenarios, the format, word count, structural design, and information volume of the news coverage remained consistent. Participants were randomly assigned to one of the scenarios and were asked to read the simulated news coverage before completing the questionnaire. A total of 592 people participated in the study online and 371 valid questionnaires were obtained after postchecking, including 88 valid questionnaires for condition 1, 99 for condition 2, 88 for condition 3, and 96 for condition 4.

**Figure 1:**
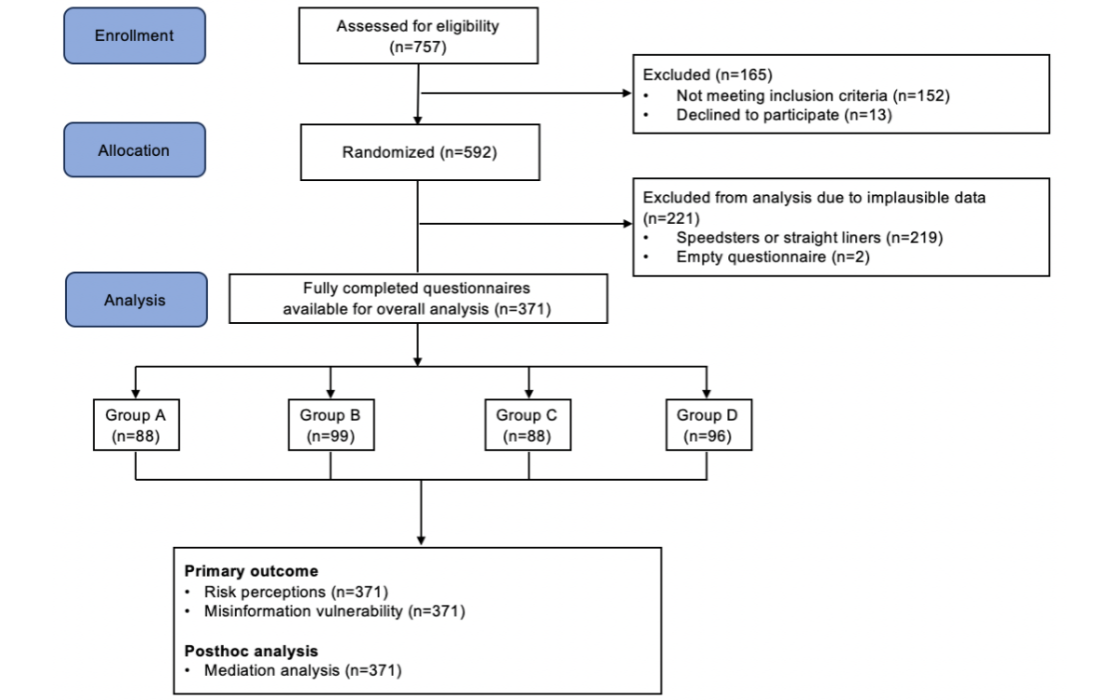
Study flow diagram.

Figure 1 shows the general flow of participants in the study. Participants were from a wide range of age groups. The sample included a relatively equal sex ratio (with 41.80% of the sample identifying as female), and the majority of the participants had an education level of college degree or above (73%). Table 2 summarizes the main sociodemographic characteristics of the sample.

**Table 1:** News article exposure simulation conditions and core messages.

Condition	Core message in simulated news article
1: Threat certainty × Solution certainty	The infectiousness of asymptomatic cases is limited and there are adequate guarantees to control the transmission range of asymptomatic cases
2: Threat certainty × Solution uncertainty	The infectiousness of asymptomatic cases is limited, but the means to control the transmission range of asymptomatic cases are uncertain
3: Threat uncertainty × Solution certainty	The infectiousness of asymptomatic cases is uncertain, but there are adequate guarantees to control the transmission range of asymptomatic cases
4: Threat uncertainty × Solution uncertainty	The infectiousness of asymptomatic cases is uncertain and the means to control the transmission range of asymptomatic cases are uncertain

**Table 2:** Sociodemographic characteristics of the participants (N=371).

Characteristics		Participants, n (%)
**Sex**		
	Male	216 (58.2)
	Female	155 (41.8)
**Education**		
	Completed PhD degree	25 (6.7)
	Completed graduate degree	88 (23.7)
	Completed college	158 (42.6)
	Completed high school	49 (13.2)
	Completed middle school	51 (13.7)
**Employment status**		
	Full-time job	167 (45.0)
	Part-time job	12 (3.2)
	Unemployed or student	176 (47.4)
	Retired	16 (4.3)
**Monthly income (US $)**		
	<428	80 (21.6)
	428-857	98 (26.4)
	857-1428	77 (20.7)
	>1428	60 (16.2)

### Ethical Considerations

The Institutional Review Board office of Renmin University of China approved the research protocol, and responses were collected via Sojump. Before the experiment, participants were asked if they agree to participate in the research voluntarily for receiving compensation. Participants received a reward of RMB 7 (US $1). The final data set is anonymized, without any identifiable private information connected to participants.

### Variables

#### Perceived Severity

Perceived severity was measured with five items adapted from a previous study [8]: (1) I think asymptomatic cases are a serious problem for us; (2) At some point in the future, we could all be threatened by asymptomatic cases; (3) I think asymptomatic cases have very serious consequences; (4) I think asymptomatic cases are a very serious problem; and (4) I think the presence of asymptomatic cases is a serious threat to my health (mean 5.26, SD 1.18; Cronbach α=0.89).

#### Perceived Susceptibility

Perceived susceptibility was measured with two items taken from an existing scale [8]: (1) I feel that I am also at risk of being infected by asymptomatic cases and (2) I feel that I may also be infected by asymptomatic cases (mean 4.86, SD 1.76; Pearson *r*=0.83, *P*<.001).

#### Information Seeking

Information seeking was measured based on the scale developed by Brashers et al [31] with the following eight items: (1) I would like to learn more information on asymptomatic cases than what is provided in this report; (2) I may discuss asymptomatic cases with people close to me (eg, friends, family); (3) I may ask my doctor about asymptomatic cases; (4) I may seek other news reports about asymptomatic cases; (5) I may pay close attention to news reports about asymptomatic cases that I encounter in the future; (6) I may check the internet for other information about asymptomatic cases; (7) I am likely to pay close attention to information on asymptomatic cases that I encounter on the internet in the future; and (8) I may read the scientific research literature on asymptomatic cases (mean 5.46, SD 1.30; Cronbach α=0.94).

#### Information Avoidance

Information avoidance was measured using the scales developed by Afifi and Weiner [13] and Evans et al [14] with the following six items: (1) I may try to change the subject if people close to me (eg, friends and family) discuss the issue of asymptomatic cases; (2) I may try to change the subject if my doctor discusses asymptomatic cases; (3) I may avoid exposure to other news reports about asymptomatic cases; (4) I may avoid exposure to information on the internet about asymptomatic cases; (5) I may try not to think too much about asymptomatic cases; and (6) I may try to forget about asymptomatic cases (mean 2.76, SD 1.73; Cronbach α=0.96).

#### Willingness to Adopt Preventive Behaviors

Willingness to adopt preventive behaviors was measured via 10 items using a 7-point Likert scale (1=strongly agree to 7=strongly disagree), which measured respondents’ willingness to adopt a range of preventive behaviors such as strengthening protection, following health instructions, trying more preventive measures, and getting vaccinated. The specific items were: (1) I have decided to strengthen my preventive measures against COVID-19 immediately; (2) I intend to strengthen my protective measures against COVID-19 in the future; (3) I will pay more attention to asymptomatic cases; (4) I will persuade people around me to pay more attention to asymptomatic cases; (5) I will strictly follow professional health instructions to prevent catching the disease; (6) I will persuade people around me to strictly follow professional health instructions to prevent disease; (7) I will try as many ways as possible to prevent disease; (8) I will persuade those around me to try as many ways as possible to prevent disease; (9) I will get vaccinated as soon as a COVID-19 vaccine is developed; and (10) I will persuade those around me to get vaccinated as soon as a COVID-19 vaccine is developed (mean 5.54, SD 1.27; Cronbach α=0.89).

#### Vulnerability to False Information

Vulnerability to false information was measured by presenting respondents with 10 pieces of false news on COVID-19 (with five real news articles provided as distractors) to measure their trust in the false news. Sample items included the following:

Isatis root is suitable for treating conditions such as the common cold and viral influenza with heat symptoms. It has an antiviral effect and can help to prevent COVID-19.

Tobacco particles are at the nanometer scale and can evenly cover the surface of lung cells, forming a barrier that keeps viruses out of the body. Therefore, smoking can reduce the infection of the novel coronavirus.

The novel coronavirus is primarily a respiratory infection virus. Gargling with saline solution can eliminate the novel coronavirus bacteria that enter through the mouth.

These items were measured on a scale of 1 (strongly disbelieve) to 10 (strongly believe) (mean 3.68, SD 1.46; Cronbach α=0.89).

### Data Analysis

To answer the four research questions, ANOVA was performed to examine the main and interaction effects of the manipulated independent variables (ie, uncertainty) on dependent outcomes (ie, risk perceptions and misinformation vulnerability). The mediation model was used as a posthoc analysis to estimate statistically significant differences between experimental conditions on outcomes. SPSS (version 28.0) and PROCESS (version 4.2) were used for all statistical analyses.

## Results

### Effects of Uncertainty Framing in News Coverage

**Figure 2:**
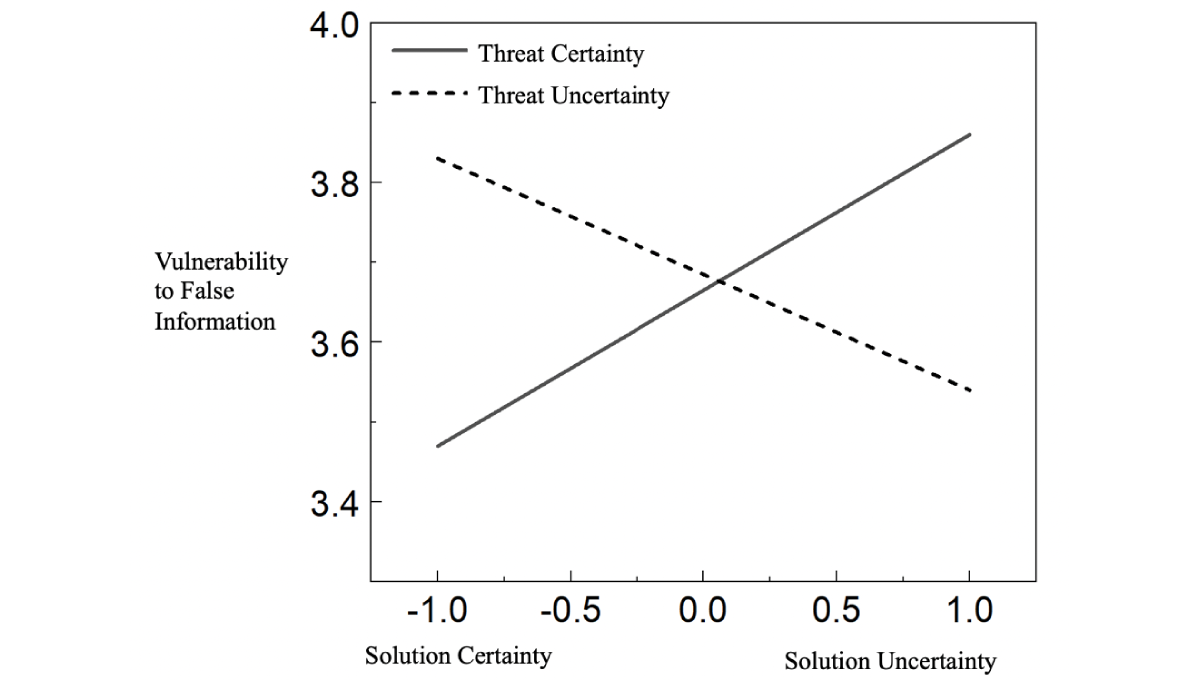
Interaction effect of an uncertainty frame of news coverage on people’s misinformation vulnerability.

According to the ANOVA results (Figure 2), there was a significant interaction effect of threat uncertainty and solution uncertainty on individuals’ vulnerability to misinformation (*F*_1, 367_=5.10, *P*=.02; partial *η^2^*=0.01). Figure 2 provides the complete data on group comparisons. Specifically, individuals who read news containing a certain threat and uncertain solution (mean 3.86, SD 0.15) were more likely to believe misinformation than those who read news containing a certain threat and certain solution (mean 3.47, SD 0.16). However, there was neither a significant main effect of threat uncertainty (partial *η^2^*=0.00) nor a significant main effect of solution uncertainty (partial *η^2^*=0.00) on individuals’ vulnerability to false information (Table 3).

In terms of information processing (Table 4), the threat uncertainty of health information showed a significant effect in promoting information-seeking behavior (partial *η^2^*=0.012). This implies that people who read news with the presence of an uncertain threat were more likely to search for additional relevant information than those who read news with absence of an uncertain threat. However, the uncertainty framing in the news, regardless of the type of uncertainty, did not affect individuals’ information avoidance behaviors.

**Table 3:** ANOVA results for determining the main effects of an uncertainty frame of a threat and solution on individuals’ risk perceptions and misinformation vulnerability.

Effect		Severity	Susceptibility	Misinformation vulnerability
**Threat**				
	Certainty, mean (SD)	5.12 (0.10)	4.86 (0.13)	3.67 (0.11)
	Uncertainty, mean (SD)	5.34 (0.10)	4.86 (0.13)	3.68 (0.11)
	*F* (*df*=1, 367)	1.68	0.00	0.01
	*P* value	.20	>.99	.91
**Solution**				
	Certainty, mean (SD)	5.10 (0.10)	4.78 (0.13)	3.65 (0.11)
	Uncertainty, mean (SD)	5.39 (0.10)	4.94 (0.13)	3.70 (0.10)
	*F* (*df*=1, 367)	4.29	0.78	0.09
	*P* value	.04	.38	.76

**Table 4:** ANOVA results for main effects of uncertainty framed in the news on motivating individuals’ information-seeking and information-avoiding behaviors.

Effect			Information seeking		Information avoidance
**Threat**					
	Certainty, mean (SD)	5.31 (0.10)		2.89 (0.13)	
	Uncertainty, mean (SD)	5.60 (0.10)		2.62 (0.13)	
	*F* (*df*=1, 367)	4.47		2.26	
	*P* value	.04		.13	
**Solution**					
	Certainty, mean (SD)	5.40 (0.10)		2.60 (0.13)	
	Uncertainty, mean (SD)	5.51 (0.10)		2.91 (0.12)	
	*F* (*df*=1, 367)	0.61		3.07	
	*P* value	.43		.08	

### Sequential Mediation Analysis

**Figure 3:**
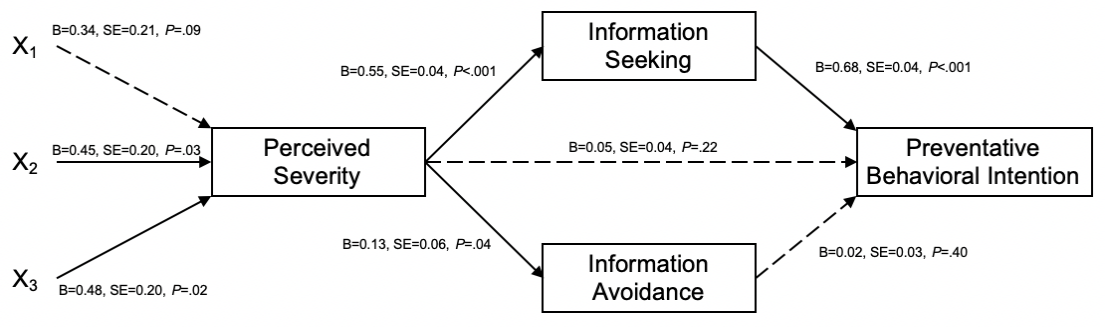
Sequential mediators of perceived severity and information processing with exposure to uncertainty for preventive behavioral intention (N=371). Significant paths are presented with solid lines and nonsignificant paths are presented with dotted lines. Dummy code: 1=uncertain threat and certain solution; 2=certain threat and uncertain solution; 3=uncertain threat and uncertain solution; and reference (0)=certain threat and certain solution.

Serial linear regression with PROCESS macro model 81 was used to analyze how the dummy-coded variables (1=uncertain threat and certain solution, 2=certain threat and uncertain solution, 3=uncertain threat and uncertain solution, and reference (0)=certain threat and certain solution) influence preventive behavioral intention through the factors perceived severity, information seeking, and information avoidance. A significant serial mediation model was detected, in which the perceived severity and information seeking would sequentially mediate the relationship between the exposure to news containing a certain threat and an uncertain solution (point estimate 0.17, SE 0.08; 95% CI 0.02-0.33) or news containing an uncertain threat and an uncertain solution (point estimate 0.18, SE 0.08; 95% CI 0.03-0.34) and the protective behavioral intention (see Figure 3 for the path significance and coefficients).

**Figure 4:**
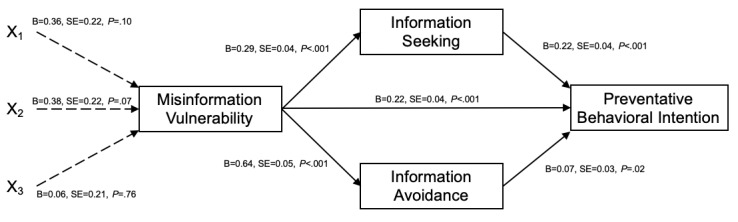
Sequential mediators of misinformation vulnerability and information processing with exposure to uncertainty for preventative behavioral intention (N=371). Significant paths are presented with solid lines and nonsignificant paths are presented with dotted lines. Dummy code: 1=uncertain threat and certain solution; 2=certain threat and uncertain solution; 3=uncertain threat and uncertain solution; and reference (0)=certain threat and certain solution.

The mediation models obtained with PROCESS macro model 81, including misinformation vulnerability, information seeking, and information avoidance as the three mediators, were established with the same three dummy-coded comparison variables described above. Although the serial mediating effect of misinformation vulnerability and information processing on the relationship was nonsignificant, significant associations between misinformation vulnerability on information processing and protective behavioral intention were detected (see Figure 4 for the path significance and coefficients).

## Discussion

### Principal Findings

This study explored the impact of incorporating different levels of uncertainty in news articles on individuals’ risk perception, susceptibility to misinformation, subsequent information processing, and intention toward preventive behaviors. These factors play a crucial role in guiding individuals to protect themselves during a public health crisis.

First, as expected, the findings showed that individuals would perceive the crisis to be of greater severity when reading news framed withing a context of solution uncertainty, regardless of whether or not the threat of the crisis was framed as certain or uncertain in the news article. Furthermore, the perceived severity would in turn motivate individuals’ information-seeking and information-avoidance behaviors. As suggested by Gudykunst [32], the experience of fear and concern is also associated with uncertainty. Individuals’ fear might be evoked when they fail to find a solution to solve the risky problem, which would consequently increase their perception of the severity of the health threat. Meanwhile, to cope with the fear, individuals would either seek more information or avoid more information [31].

Second, individuals tended to believe false information during a public health crisis, especially after reading a news article containing certain threats and uncertain solutions. Furthermore, posthoc regression analysis suggested that both information seeking and information avoidance were positively associated with misinformation vulnerability. As expected, information seeking was positively associated with protective behavioral intentions, while information avoidance was negatively associated with protective behavioral intention. That said, communicating crisis uncertainty in a news article would be risky in terms of increasing the public’s vulnerability to misinformation. Moreover, misinformation vulnerability would further motivate information avoidance, which would consequently dissuade individuals’ intention to adopt preventive behaviors. However, communicating crisis uncertainty could also be beneficial because the increased misinformation vulnerability that arises after reading news with related uncertainty could simultaneously motivate both expected health information–seeking and protective behaviors.

Third, our findings suggest that compared to an uncertain threat, uncertain solutions are more potentially problematic. An uncertain solution with either an uncertain or certain threat had a greater impact on individuals’ perceived severity of the crisis. Moreover, an uncertain solution with an uncertain threat led to higher vulnerability to misinformation. Although both perceived severity and misinformation vulnerability could motivate expected health information–seeking or protective behavioral intentions, they were likely to trigger information avoidance, which would further impair the protective behavioral intention. Given these conflicting findings, more research is needed to understand the mechanism behind the effects of communicating solution uncertainty, especially during a public health crisis.

### Implications and Limitations

A few limitations of this study should be noted to provide inspiring suggestions for further research. First, the generalizability of the findings needs to be addressed. This study is based on an exceptionally unique context of a public health event. In April 2020, the ambiguity surrounding the perceived risk of COVID-19 and the appropriate preventive measures was prevalent. Different regions in China implemented varying degrees of epidemic control measures, leading to divergent strategies. Media coverage of “asymptomatic carriers” and other aspects of the COVID-19 pandemic often exhibited inconsistent or contradictory information. Thus, the diverse experimental scenarios were based on natural contexts for presenting four distinct threat-and-solution scenarios and would not induce a perceptual conflict for the participants. However, when extrapolating to other research topics, it is imperative to consider the coherence between experimental scenarios and real-world settings.

Second, the impact of the research topic on participants with different characteristics requires further discussion. This study endeavored to achieve a balanced representation of participants with respect to sex, education level, and income bracket. Consequently, an exhaustive examination of the differential effects stemming from various sociodemographic factors on the outcomes was not performed. However, in distinct research inquiries, this divergence may prove consequential. Hence, future research endeavors could delve deeper into understanding the perception of information uncertainty and management behaviors across populations with diverse backgrounds and characteristics.

Third, although this type of experimental design typically includes 80-90 participants per group to achieve sufficient statistical power, the unexpected smaller effect size in this study resulted in a reduced statistical power of approximately 60%. This diminutive effect size may compromise the study’s sensitivity in detecting meaningful relationships, thereby affecting the reliability and generalizability of the findings. To enhance statistical power and ensure robust results in similar experimental studies in China, a larger sample size is recommended.

Regarding the implications, this study aimed to elucidate the impact of health information uncertainty on individuals’ information-processing mechanisms. The results thus provide further evidence for the impacts of individuals’ perceptions and behaviors underlying uncertainty management theory. Amid public health events inundated with uncertain information, individuals’ perceptions and behaviors related to uncertainty management often determine their attitudes toward addressing health threats and the potential adoption of health measures. Therefore, comprehending this process contributes to facilitating more effective health communication between the public health system and the general public. Faced with uncertain public health events, participants such as public health institutions, media, and the general public should all take into account the implications of information uncertainty, ensuring the effective dissemination of information throughout all stages of the crisis.

## Appendix

Multimedia Appendix 1 Simulated news articles presented to the participants in the online experiment under four conditions varying in threat and solution uncertainty about the COVID-19 pandemic.
