# Supplementary material for: Understanding the Impact of Communicating Uncertainty About COVID-19 in the News: Randomized Between-Subjects Factorial Experiment
Source: J Med Internet Res. 2024 May 14;26:e51910. doi: 10.2196/51910 (PMC11095512; doi:10.2196/51910)
Supplement: Multimedia Appendix 1 [file jmir_v26i1e51910_app1.docx]

Experimental Materials

Condition 1


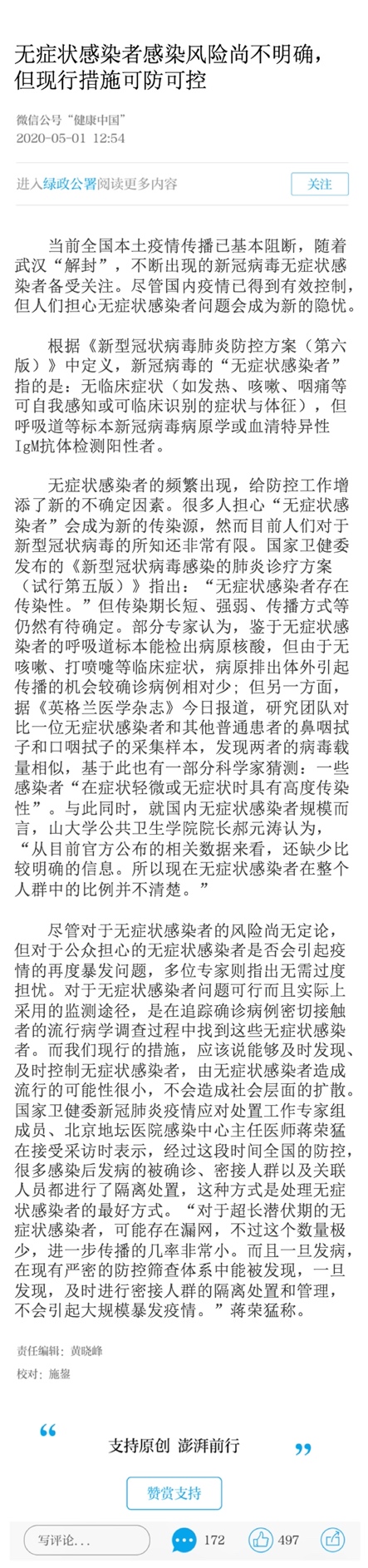


*Title: The infectiousness of asymptomatic cases is limited, and there are adequate guarantees to control the transmission range of asymptomatic cases*

As the nationwide spread of the local COVID-19 outbreak has been largely contained, the emergence of asymptomatic carriers following the "unsealing" of Wuhan has raised concerns. Despite effective control measures, people worry that asymptomatic carriers could become a new hidden threat.

According to the "Novel Coronavirus Pneumonia Prevention and Control Plan (Sixth Edition)," asymptomatic carriers of the novel coronavirus are defined as individuals showing no clinical symptoms (such as fever, cough, sore throat, etc., that are self-perceivable or clinically identifiable), but testing positive for the novel coronavirus in respiratory tract specimens or with specific IgM antibodies in serum. Liu Youning, former director of the Institute of Respiratory Diseases of the People's Liberation Army, explained in an interview that confirmed cases without symptoms can be classified into two types: those who remain asymptomatic after a 14-day incubation period and those who, although asymptomatic during sampling, later show clinical symptoms, being in a "asymptomatic infection" state during the incubation period.

The frequent occurrence of asymptomatic carriers has introduced new uncertainties into preventive efforts. Many are concerned that asymptomatic carriers could become new sources of infection, yet the understanding of the novel coronavirus is still limited. The "Diagnosis and Treatment Plan for Novel Coronavirus Infection Pneumonia (Fifth Trial)" released by the National Health Commission states, "Asymptomatic carriers can be infectious," but the duration, strength, transmission methods, etc., are still to be determined.

Some experts suggest that, since respiratory specimens of asymptomatic carriers can detect viral nucleic acids, but with a lower chance of spreading due to the absence of clinical symptoms like coughing or sneezing, the transmission risk is relatively lower compared to confirmed cases. However, a report from the "British Medical Journal" today suggests that the virus load in samples from an asymptomatic carrier and an ordinary patient is similar. Based on this, some scientists speculate that some carriers may have "high infectivity during mild or asymptomatic periods." Meanwhile, regarding the scale of asymptomatic carriers in China, Hao Yuantao, dean of the School of Public Health at Shandong University, believes, "From the currently released official data, there is still a lack of clear information. Therefore, the proportion of asymptomatic carriers in the entire population is unclear."

The identification and control of asymptomatic carriers, as well as addressing the risk of transmission by asymptomatic carriers after resuming work and production, are topics of extensive discussion. On the one hand, the hidden nature of asymptomatic carrier transmission poses a challenge because their lack of noticeable external symptoms makes it difficult to identify them from the healthy population immediately, potentially leading to widespread virus dissemination without their knowledge. On the other hand, subjective symptoms of asymptomatic carriers may lead them to believe they are not infected, making it challenging to detect them in routine medical work. Therefore, unless a large-scale population screening is conducted, it is difficult to identify all asymptomatic carriers after resuming work. However, due to the testing window period, using nucleic acid and serological testing methods is challenging to detect all asymptomatic carriers, and mass specimen testing requires substantial human and material resources, making it difficult to achieve. Therefore, finding a consensus on addressing the threat of asymptomatic carriers to COVID-19 prevention and control requires joint efforts from relevant medical institutions and experts to actively seek solutions

Condition 2


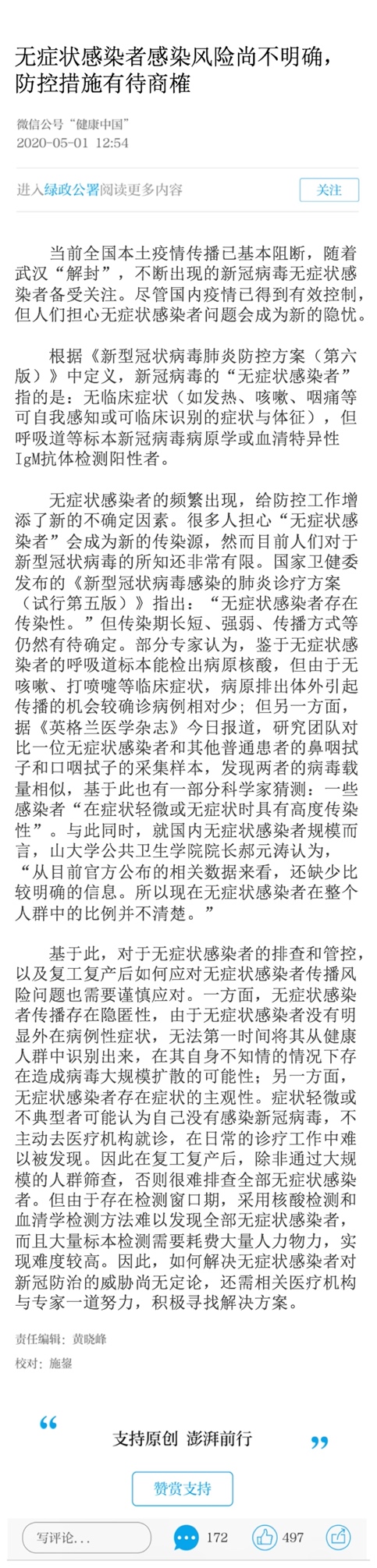


*Title: The infectiousness of asymptomatic cases is limited, but the means to control the transmission range of asymptomatic cases are uncertain*

As the nationwide spread of the local COVID-19 outbreak has been largely contained, the emergence of asymptomatic carriers following the "unsealing" of Wuhan has raised concerns. Despite effective control measures, people worry that asymptomatic carriers could become a new hidden threat.

According to the "Novel Coronavirus Pneumonia Prevention and Control Plan (Sixth Edition)," asymptomatic carriers of the novel coronavirus are defined as individuals showing no clinical symptoms but testing positive for the virus in respiratory tract specimens or with specific IgM antibodies in serum. Liu Youning, former director of the Institute of Respiratory Diseases of the People's Liberation Army, explained that those confirmed as "infected" but asymptomatic can be classified into two types: those who remain asymptomatic after a 14-day incubation period and those who, although asymptomatic during sampling, later show clinical symptoms, being in an "asymptomatic infection" state during the incubation period.

The frequent appearance of asymptomatic carriers has introduced new uncertainties into preventive efforts. On February 3rd, the National Health Commission explicitly stated in the "Diagnosis and Treatment Plan for Novel Coronavirus Infection Pneumonia (Fifth Trial)" that "asymptomatic carriers can also become sources of transmission." However, addressing public concerns about whether asymptomatic carriers could trigger a resurgence of the epidemic, Chinese Academy of Engineering academician Zhong Nanshan stated to the media on the 29th that the recent number of newly confirmed COVID-19 cases in China has not only not increased but is continuously decreasing. This suggests that there are not a large number of "asymptomatic carriers" in China. Jiang Qingwu, former dean of the School of Public Health at Fudan University, holds a similar view, affirming the existence of covert infections but asserting that the proportion is not high and has minimal impact on current prevention and control measures. Regarding transmission capability, the common characteristic of respiratory infectious diseases is that patients become more contagious as they develop a fever and cough more. Sneezing produces droplets, and fever is a result of the virus replicating extensively in the body. Asymptomatic carriers, lacking these direct signs, generally have a weaker virus transmission capability compared to symptomatic individuals. However, caution is still necessary from an epidemic prevention perspective.

Additionally, several experts pointed out that the current monitoring approach for asymptomatic carriers, both in screening and controlling the spread after resuming work and production, involves finding these carriers during the epidemiological investigation of close contacts of confirmed cases. Jiang Rongmeng, a member of the National Health Commission's expert group on COVID-19 response and director of the Infection Center at Beijing Ditang Hospital, stated in an interview that the current measures are effective in promptly identifying and controlling asymptomatic carriers. The likelihood of asymptomatic carriers causing widespread transmission at the societal level is minimal. After weeks of nationwide prevention and control efforts, isolation measures have been implemented for diagnosed cases, close contacts, and associated individuals. This approach is the best way to handle asymptomatic carriers. Jiang Rongmeng further mentioned that although there may be a small number of asymptomatic carriers with an extended incubation period, the probability of further transmission is very low. Once symptoms appear, the existing rigorous prevention and control screening system can detect them, leading to prompt isolation and management of close contacts, preventing the occurrence of a large-scale epidemic.

Condition 3


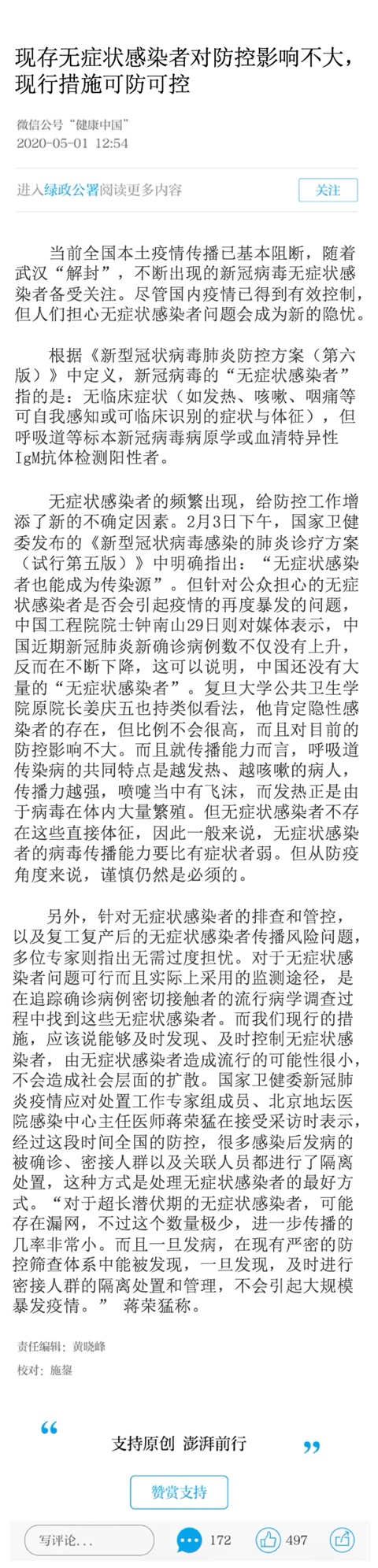


*Title: The infectiousness of asymptomatic cases is uncertain, but there are adequate guarantees to control the transmission range of asymptomatic cases*

As the nationwide spread of the local COVID-19 outbreak has been largely contained, the emergence of asymptomatic carriers following the "unsealing" of Wuhan has raised concerns. Despite effective control measures, people worry that asymptomatic carriers could become a new hidden threat.

According to the "Novel Coronavirus Pneumonia Prevention and Control Plan (Sixth Edition)," asymptomatic carriers of the novel coronavirus are defined as individuals showing no clinical symptoms but testing positive for the virus in respiratory tract specimens or with specific IgM antibodies in serum. Liu Youning, former director of the Institute of Respiratory Diseases of the People's Liberation Army, explained that those confirmed as "infected" but asymptomatic can be classified into two types: those who remain asymptomatic after a 14-day incubation period and those who, although asymptomatic during sampling, later show clinical symptoms, being in an "asymptomatic infection" state during the incubation period.

The frequent appearance of asymptomatic carriers has introduced new uncertainties into preventive efforts. Many worry that asymptomatic carriers could become new sources of infection. However, the current understanding of the novel coronavirus is limited. The "Diagnosis and Treatment Plan for Novel Coronavirus Infection Pneumonia (Fifth Trial)" released by the National Health Commission points out that "asymptomatic carriers are infectious." However, details such as the duration, strength, and mode of transmission of infectivity are still pending confirmation. Some experts believe that, considering the ability to detect viral nucleic acids in respiratory specimens of asymptomatic carriers but with a lower chance of transmission due to the absence of clinical symptoms like coughing or sneezing, the spread risk is relatively lower compared to confirmed cases. However, a recent report from the "British Medical Journal" suggests that virus loads in samples from asymptomatic carriers and ordinary patients are similar, leading some scientists to speculate that some carriers "may have high infectivity during mild or asymptomatic periods." Meanwhile, regarding the scale of asymptomatic carriers in China, Hao Yuantao, dean of the School of Public Health at Shandong University, believes, "From the currently released official data, there is still a lack of clear information. Therefore, the proportion of asymptomatic carriers in the entire population is unclear."

Although the risk of asymptomatic carriers is not definitively established, experts point out that the current monitoring approach involves finding these carriers during the epidemiological investigation of close contacts of confirmed cases. The existing measures are effective in promptly identifying and controlling asymptomatic carriers. The likelihood of asymptomatic carriers causing widespread transmission at the societal level is minimal. Jiang Rongmeng, a member of the National Health Commission's expert group on COVID-19 response and director of the Infection Center at Beijing Ditang Hospital, stated in an interview that after weeks of nationwide prevention and control efforts, diagnosed cases, close contacts, and associated individuals have undergone isolation measures. This approach is the best way to handle asymptomatic carriers. Jiang Rongmeng further mentioned that although there may be a small number of asymptomatic carriers with an extended incubation period, the probability of further transmission is very low. Once symptoms appear, the existing rigorous prevention and control screening system can detect them, leading to prompt isolation and management of close contacts, preventing the occurrence of a large-scale epidemic.

Condition 4


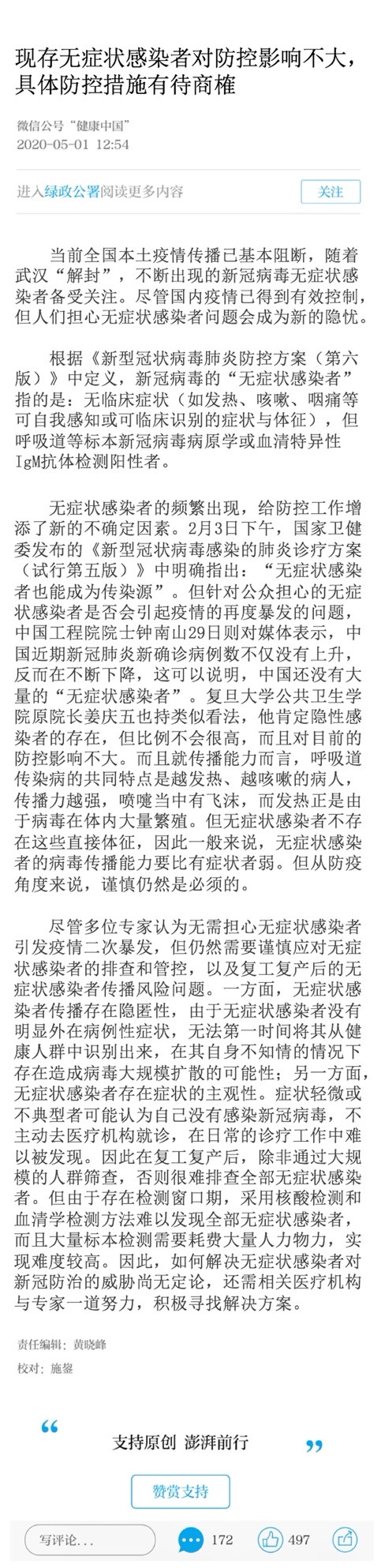


*Title: The infectiousness of asymptomatic cases is uncertain, the means to control the transmission range of asymptomatic cases are uncertain*

With the nationwide spread of local COVID-19 cases being largely contained, the emergence of asymptomatic carriers of the new coronavirus following the "unsealing" of Wuhan has attracted attention. Despite effective control of the domestic epidemic, concerns have arisen about asymptomatic carriers becoming a new source of worry.

As defined by the "Novel Coronavirus Pneumonia Prevention and Control Plan (Sixth Edition)," asymptomatic carriers of the novel coronavirus exhibit no clinical symptoms (such as fever, cough, or throat discomfort) but test positive for the virus in respiratory tract specimens or show specific IgM antibodies in serum. Liu Youning, former director of the Institute of Respiratory Diseases of the People's Liberation Army, explained in an interview that individuals confirmed as "infected" but asymptomatic can be classified into two categories: those with positive nucleic acid tests who remain asymptomatic after a 14-day observation period and those with positive nucleic acid tests who, during sampling, show no symptoms but later develop some clinical manifestations, placing them in a state of "asymptomatic infection" during the incubation period.

The frequent appearance of asymptomatic carriers has introduced new uncertainties into prevention and control efforts. In the afternoon of February 3, the "Diagnosis and Treatment Plan for Novel Coronavirus Infection Pneumonia (Fifth Trial)" issued by the National Health Commission explicitly stated, "Asymptomatic carriers can also become sources of transmission." Addressing concerns among the public about whether asymptomatic carriers could lead to a second outbreak of the epidemic, Chinese Academy of Engineering academician Zhong Nanshan stated to the media on the 29th that the number of newly diagnosed cases of COVID-19 in China has not only failed to rise but is continuously decreasing. This suggests that there are not a large number of "asymptomatic carriers" in China. Jiang Qingwu, former dean of the School of Public Health at Fudan University, shares a similar view, affirming the existence of asymptomatic carriers but stating that their proportion is not high, and they have minimal impact on current prevention and control measures. Additionally, in terms of transmission capability, the common characteristic of respiratory infectious diseases is that patients become more infectious with fever and coughing, as these actions release droplets. Fever occurs because the virus replicates extensively within the body. However, asymptomatic carriers lack these direct signs. Generally, the virus transmission capability of asymptomatic carriers is weaker than that of symptomatic individuals. Nonetheless, from the perspective of epidemic prevention, caution is still necessary.

While several experts believe there is no need to worry about asymptomatic carriers triggering a second outbreak, cautious measures are still required for the detection and control of asymptomatic carriers, especially concerning the potential risk of transmission after resuming work and production. On the one hand, the transmission of asymptomatic carriers is covert, as they lack obvious external symptoms, making it challenging to identify them promptly among the healthy population. This poses a potential risk of large-scale virus spread unknowingly. On the other hand, the subjectivity of symptoms among asymptomatic carriers complicates the situation. Those with mild or atypical symptoms may believe they are not infected with the novel coronavirus, avoiding seeking medical attention and making it challenging to be detected in routine medical work. Therefore, after the resumption of work and production, unless through large-scale population screening, it is difficult to identify all asymptomatic carriers. However, due to the existence of the testing window period, it is challenging to detect all asymptomatic carriers using nucleic acid and serological testing methods. Moreover, extensive sample testing requires significant human and material resources, making implementation more difficult. Therefore, finding a consensus on addressing the threat of asymptomatic carriers to COVID-19 prevention and treatment requires collaborative efforts from relevant medical institutions and experts to actively seek solutions.
